# HIV-1 and methamphetamine co-treatment in primary human astrocytes: TAARgeting ER/UPR dysfunction

**DOI:** 10.1515/nipt-2023-0020

**Published:** 2024-02-19

**Authors:** Jessica M. Proulx, In-Woo Park, Kathleen Borgmann

**Affiliations:** Department of Microbiology, Immunology and Genetics at University of North Texas Health Science Center, Fort Worth, TX, 76107, USA; Sanford Burnham Prebys Medical Discovery Institute, La Jolla, CA, 92037, USA; National Institute on Drug Abuse, North Bethesda, MD, 20852, USA

**Keywords:** astrocytes, HIV-associated neurocognitive disorders (HAND), methamphetamine (METH), unfolded protein response (UPR), calcium signaling, mitochondria-associated ER membranes (MAMs)

## Abstract

**Objectives:**

Human immunodeficiency virus 1 (HIV-1) can invade the central nervous system (CNS) early during infection and persist in the CNS for life despite effective antiretroviral treatment. Infection and activation of residential glial cells lead to low viral replication and chronic inflammation, which damage neurons contributing to a spectrum of HIV-associated neurocognitive disorders (HAND). Substance use, including methamphetamine (METH), can increase one’s risk and severity of HAND. Here, we investigate HIV-1/METH co-treatment in a key neurosupportive glial cell, astrocytes. Specifically, mitochondria-associated endoplasmic reticulum (ER) membrane (MAM) signaling pathways, such as calcium and the unfolded protein response (UPR), are key mechanisms underlying HAND pathology and arise as potential targets to combat astrocyte dysfunction.

**Methods:**

Primary human astrocytes were transduced with a pseudotyped HIV-1 model and exposed to low-dose METH for seven days. We assessed changes in astrocyte HIV-1 infection, inflammation, mitochondrial antioxidant and dynamic protein expression, respiratory acitivity, mitochondrial calcium flux, and UPR/MAM mediator expression. We then tested a selective antagonist for METH-binding receptor, trace amine-associated receptor 1 (TAAR1) as a potetnial upstream regulator of METH-induced calcium flux and UPR/MAM mediator expression.

**Results:**

Chronic METH exposure increased astrocyte HIV-1 infection. Moreover, HIV-1/METH co-treatment suppressed astrocyte antioxidant and metabolic capacity while increasing mitochondrial calcium load and protein expression of UPR messengers and MAM mediators. Notably, HIV-1 increases astrocyte TAAR1 expression, thus, could be a critical regulator of HIV-1/METH co-treatment in astrocytes. Indeed, selective antagonism of TAAR1 significantly inhibited cytosolic calcium flux and induction of UPR/MAM protein expression.

**Conclusion:**

Altogether, our findings demonstrate HIV-1/METH-induced ER-mitochondrial dysfunction in astrocytes, whereas TAAR1 may be an upstream regulator for HIV-1/METH-mediated astrocyte dysfunction.

## Introduction

**Figure 1:**
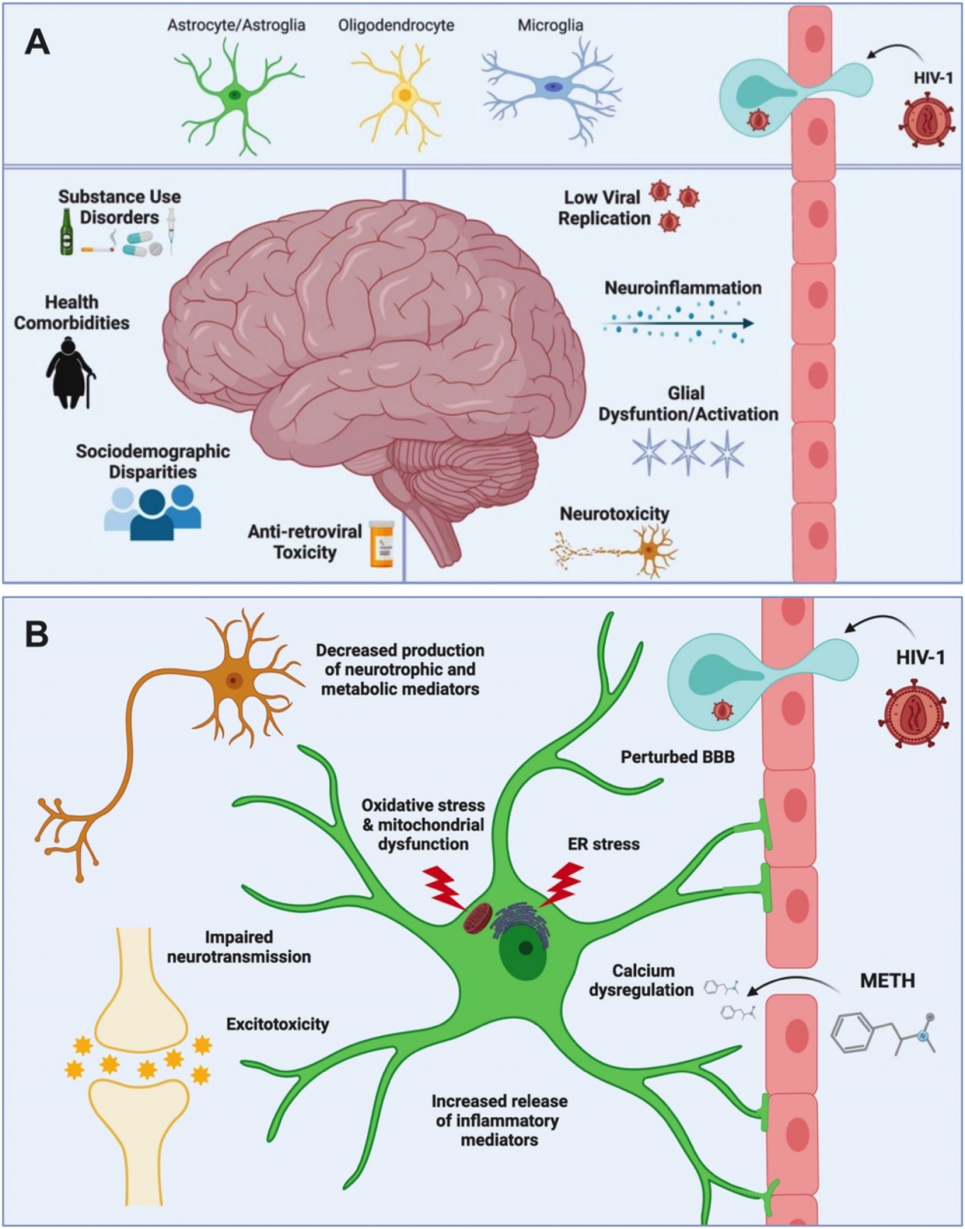
HAND pathology and astrocyte dysfunction. (A) The pathology of HAND is characterized by the early invasion of HIV-1 into the CNS mediated by infiltrating infected immune cells where it can then infect residential glial cells (including astrocytes, microglia, and oligodendrocytes) and persists in the CNS for life. Chronic levels of low viral replication, neuroinflammation, glial dysfunction/activation, and neurotoxicity are the key biological characteristics perpetuating the clinical manifestation of HAND. However, there are also additional factors that influence both the development and severity of HAND, including ART toxicity, sociodemographic disparities, health comorbidities, substance use disorders. (B) During a neuropathological challenge, astrocytes become activated, shifting their primary neurosupportive functions. During acute insult or injury (such as stroke), these functional changes can be neuroprotective; however, chronic astrocyte activation during conditions like HIV-1 infection or METH use disorders leads to astrocyte dysfunction which can mediate neurotoxic consequences. These functional outcomes include ER and oxidative stress; mitochondrial dysfunction; calcium dysregulation; increased inflammatory profile; impaired neurotransmission and tripartite synaptic maintenance; glutamate excitotoxity; decreased neurotrophic, antioxidant and metabolite support, increased release of toxic ROS/RNS radical and/or extracellular ATP, and perturbed BBB integrity. Indeed, these characteristics of astrocyte dysfunction and astrocyte-mediated neurotoxicity are hallmarks of neurodegenerative pathologies. Identifying cellular and/or molecular targets to prevent astrocyte dysfunction and optimize the coupling between astrocytes and neurons is essential to promote neuronal fitness during a neuropathological challenge. Image made with BioRender. BBB, blood-brain barrier; ER, endoplasmic reticulum; HIV-1, human immunodeficiency virus type 1; METH, methamphetamine.

There are approximately 38 million people living with HIV-1 (PLWH) worldwide. Despite effective antiretroviral therapy, approximately 50 % of PLWH develop some form of HIV-associated neurocognitive disorders (HAND) [1, 2]. The spectra of HAND are caused by early invasion of HIV-1 into the central nervous system (CNS) leading to persistent low-level viral replication, chronic neuroinflammation, glial cell dysfunction and neurotoxicity. The development and/or severity of HAND is further compounded by ART toxicity, socioeconomic factors, health comorbidities, and substance use disorders (Figure 1A). According to the National Institute on Drug Abuse (NIDA), substance use disorders, including methamphetamine (METH), are a common comorbidity among PLWH. One 2020 study found that as many as 1 in 3 new HIV transmissions among sexual and gender minorities involve people who regularly use METH [3]. Thus, NIDA has declared the intersection of HIV-1 and substance use as a research priority. Briefly, the use of METH can induce neurotoxic and neurodegenerative consequences including increased blood-brain barrier (BBB) permeability, neuroinflammation, excitotoxicity, oxidative and endoplasmic reticulum (ER) stress, calcium dysregulation, and mitochondrial dysfunction which can increase one’s risk and severity of HAND [4–6].

Astrocytes are central mediators in both CNS homeostasis and neuroinflammation, implicating them in both HIV-1 and METH neuropathology [7, 8]. In fact, astrocyte dysfunction is a hallmark of neurodegenerative pathologies [9]. Astrocytes are a major glial cell in the CNS and are fundamental for neuronal support. Their foot processes are critical in maintaining the BBB integrity as well as neurosynaptic communication through the ‘tripartite synapse’. Moreover, astrocytes provide essential metabolic, antioxidant, and neurotrophic support to neurons to ensure neuronal health and function. During a neuropathological challenge, such as HIV-1 infection or METH exposure, astrocytes can shift their neuroprotective functions and instead become neurotoxic (Figure 1B) [4, 7, 10], [11], [12], [13], [14], [15], [16], [17]. For example, astrocyte activation by HIV-1 or METH has been characterized by an altered metabolic [10, 11, 17] and/or inflammatory profile [10, 18], which threatens the provision of essential neurotrophic factors, including metabolite and antioxidant support. Likewise, astrocytes increase release of neurotoxic factors including excessive ATP [19, 20], toxic radicals [10, 20], and inflammatory cytokines [10, 18].

By elucidating the intracellular mechanisms governing human astrocyte dysfunction during concomitant METH and HIV-1 exposure, better therapeutic targets can be identified to ameliorate astrocyte-associated neurotoxicity. It is well-established that the ER and mitochondria maintain constant communication, including direct contact sites termed mitochondria-associated ER membranes (MAMs) [21, 22]. Prior studies of ER/mitochondrial cooperation have emphasized the ER as a regulator of mitochondrial function via calcium signaling and the unfolded protein response (UPR), especially under stress [16, 17, 22], [23], [24]. Indeed, HIV-1 relevant stimuli or METH exposure can induce ER stress in astrocytes [25–28] as well as alter mitochondrial function, health, and/or morphology [11–13]. Notably, at least two METH receptors have been identified in astrocytes, trace amine associated receptor 1 (TAAR1) [7, 15, 29] and sigma 1 receptor (Sig1R) [30, 31], which have been shown to regulate METH-associated astrocyte dysfunction. Identifying cellular or molecular targets that regulate astrocyte neuroprotective versus neurotoxic are critical to restore a neurosupportive phenotype to protect neurons during CNS pathologies.

The current report is focused on effects of low-dose chronic METH exposure on astrocyte HIV-1 infection and how HIV-1/METH co-treatment impacts the function and physiology of astrocyte ER and mitochondrial homeostasis. We then explore TAAR1, a METH-binding receptor in astrocytes that is upregulated by HIV-1, as a potential upstream regulator of METH-mediated astrocyte ER/UPR dysfunction [15, 29].

## Methods

### Primary human astrocyte cultures

All experiments were performed in primary human astrocytes in full compliance with local, federal, and NIH ethical guidelines. Human fetal brain tissues from first or second trimester were provided by a biorepository at the University of Washington with written informed consent obtained from all donors. Astrocyte cultures were isolated and characterized as previously described [14, 32]. Fresh and cryopreserved astrocyte cultures were used experimentally between passages two and seven. A total of 21 human astrocytes donors were isolated and evaluated for the present investigations. The biological sex and age of donors are illustrated in Table 1. Approximately six males and 13 females were tested with two donors of unknown sex. All experiments were replicated in three or more astrocyte cultures isolated from biologically distinct biospecimens.

**Table 1:** Demographic information of donor astrocytes used for HIV-1/METH co-treatment studies. (F, female; M, male; Unk, unknown).

Donor #	Sex	Gestational age	Figure(s) used
1	F	87 d	Figures 2, 3, and 4
2	F	108 d	Figures 2, 3, and 4
3	Unk	125 d	Figures 2, 3, 5, 6, and Supplementary Figure 2
4	F	89 d	Figures 2, 4, 5, and Supplementary Figure 1
5	M	89 d	Figures 2, 4 and 6
6	F	108 d	Figures 2 and 5
7	F	96 d	Figures 2 and 5
8	F	90 d	Figures 2 and 5
9	F	105 d	Figures 2 and 6
10	M	120 d	Figure 3
11	M	89 d	Figure 3
12	M	113 d	Figure 3
13	F	74 d	Figure 3
14	F	125 d	Figures 3 and 5
15	F	113 d	Figure 6
16	F	127 d	Figure 6
17	F	122 d	Figure 6
18	M	89 d	Figure 6
19	F	101 d	Figure 6, and Supplementary Figure 2
20	M	125 d	Supplementary Figure 2
21	Unk	127 d	Supplementary Figure 2

Additional methods and references are available in Supplemental Materials.

## Results

**Figure 2:**
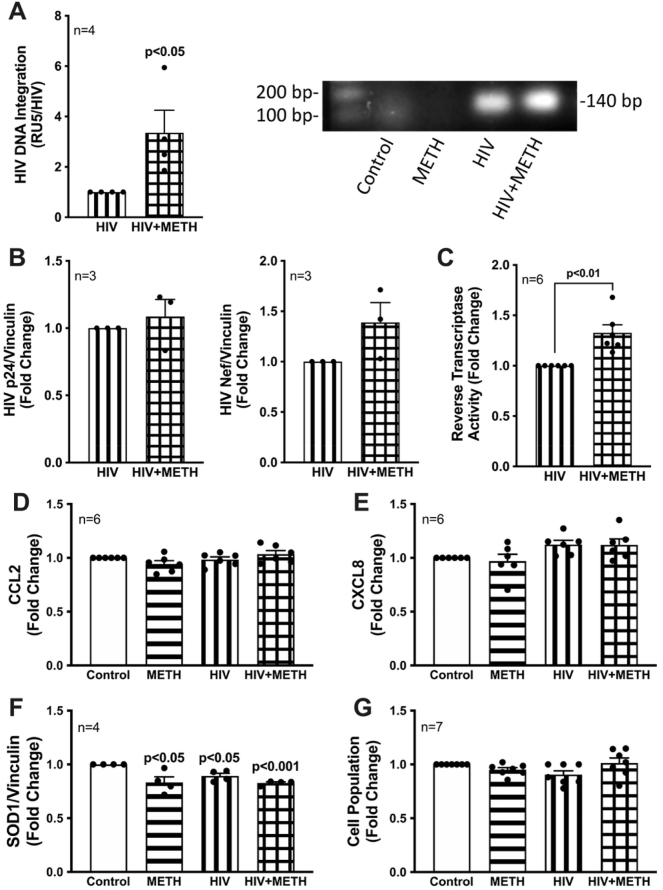
METH exposure increases astrocyte HIV-1 infection and HIV/METH impair astrocyte antioxidant support. Astrocytes were treated with METH (50 nM; horizontal lines), transduced with pseudotyped HIV-1 (500 RT; vertical lines), or co-treated with METH and pseudotyped HIV-1 (checkered lines) for seven days. (A) HIV-1 DNA integration was detected using a two-step Alu-Gag PCR assay. The amplified HIV-1 RU5 specific regions were quantified by RTPCR prior to agarose DNA gel electroporation. (B) Detection of HIV-1 proteins p24 or Nef in lysates were measured using Simple Wes. Vinculin was used as an internal control for lysates. (C) Extracellular viral production in cell supernatants was measured via radiometric reverse transcriptase (RT) activity assay. (D) CCL2 and (E) CXCL8 levels in supernatants were assessed by ELISA. (F) Protein expression of SOD1 was measured via Simple Wes. (G) Cell populations were counted at 5 d passages prior to plating for other assays. Fold changes for DNA integration and HIV-1 proteins were calculated to HIV-1 and statistical significance was determined via two-tailed paired *t* test. Fold changes for CCL2, CXCL8, SOD1 protein and cell number levels were calculated to control, and statistical significance was determined via one-way ANOVA followed by Fisher’s LSD test for multiple comparisons. Experiments were performed in a minimum of three distinct human astrocyte cultures, each represented by a dot in bar graphs (n). CCL2, C-C motif chemokine ligand 2; CXCL8, C-X-C motif chemokine ligand 8; HIV-1, human immunodeficiency virus type 1; METH, methamphetamine; Nef, negative factor; SOD1, superoxide dismutase 1.

Previous reports on HIV-1/METH comorbidity demonstrate METH exposure can impair immune function and therapy efficacy while also enhancing viral replication and infectivity [4, 33], [34], [35]. Astrocytes do not express the key receptor (CD4) required for conventional HIV-1 entry. However, astrocytes can undergo other means of HIV-1 infection such as direct cell-cell transfer via infected CD4+ T cells [36, 37], whose trafficking in to the CNS has been established [38, 39]. Therefore, to specifically investigate the consequences of METH exposure on astrocyte HIV-1 infection and inflammation, a pseudotyped HIV-1 that modifies the viral coat with vesicular stomatitis virus glycoprotein (VSVg) permitted entry independent of CD4 expression as previously described [14]. A T-tropic HIV-1 strain (NL4-3) was used to model astrocytes infected *in vivo* by T-cell-mediated HIV-1 transfer. Astrocytes were transduced with pseudotyped HIV-1 for seven days to better understand the effects of HIV-1 on astrocyte biology. Viral construct and concentration were optimized for HIV-1 DNA integration, detectable viral protein expression, and minimal cytotoxicity to best mimic chronic *in vivo* HIV-1 astrocyte infection. To recapitulate the intermittent low levels of METH in the CNS between binges of a regular user, astrocytes were exposed to low dose METH (50 nM) for seven days (horizontal lines), which is slightly below the prolonged METH ranges (60–600 nM) found *in vivo* (see Supplementary Materials and Methods). Pseudotyped HIV-1 transduction alone (500 RT; vertical lines) or in combination METH exposure for seven days (checkered lines), revealed METH-induced increases in HIV-1 DNA integration (Figure 2A; 4-fold; p<0.05), expression of HIV-1 proteins (Figure 2B), p24 (10 %; not significant), nef (40 %; not significant), and detection of HIV-1 production via reverse transcriptase activity in culture supernatants (Figure 2C; 25 %; p<0.05). There was no effect on astrocyte inflammatory mediators, CCL2 (Figure 2D) or CXCL8 (Figure 2E) in our model for chronic METH exposure and/or HIV-1 infection. However, protein expression of superoxide dismutase 1 (SOD1), a key antioxidant produced by astrocytes to detoxify oxidative radicals in the CNS, was significantly decreased following seven days of METH exposure or HIV-1 transduction, whether alone (p<0.05) or in combination (p<0.001) (Figure 2F). These treatments did not alter changes in astrocyte cell growth (Figure 2G).

**Figure 3:**
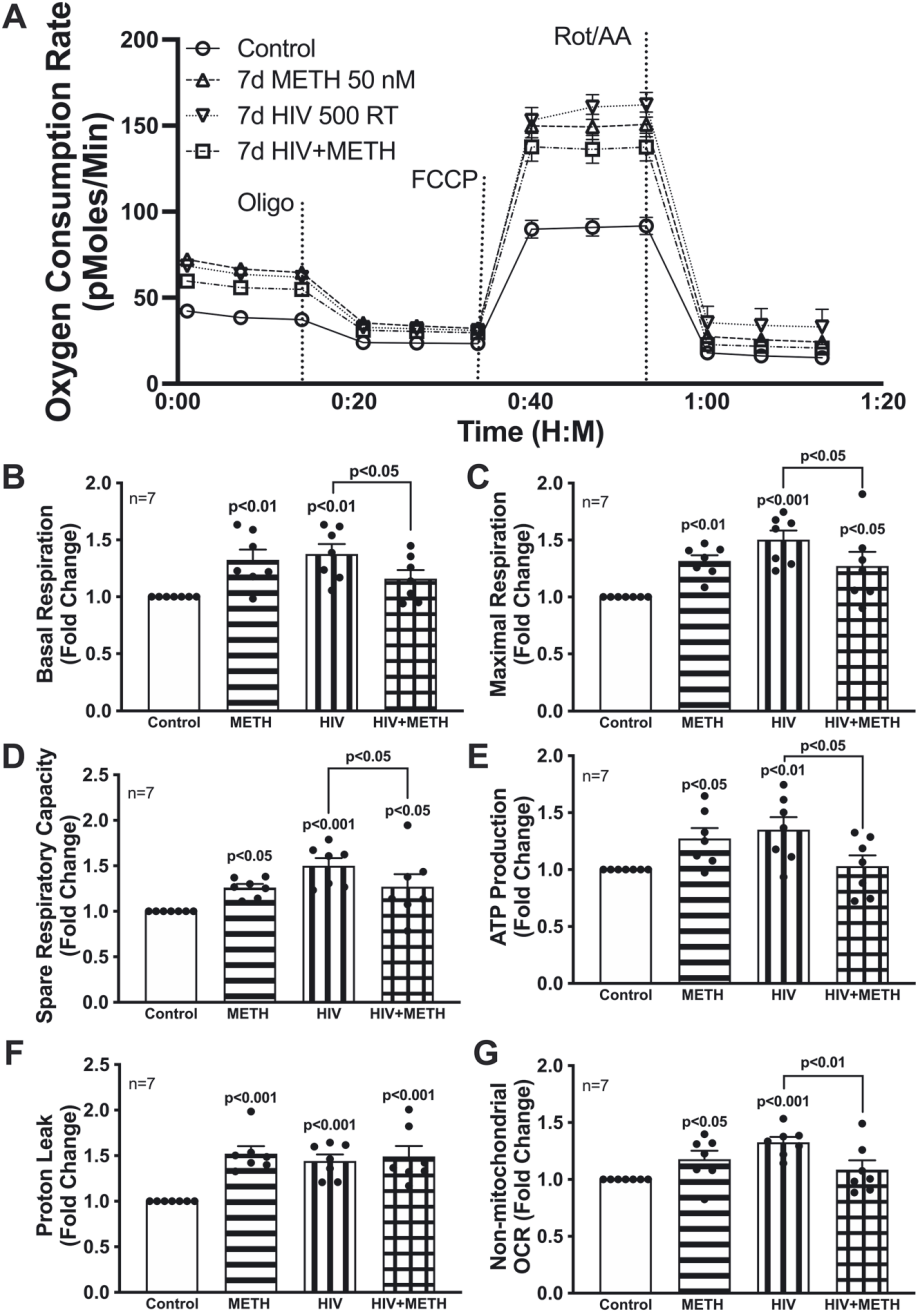
METH exposure and HIV-1 transduction increase astrocyte respiration, which is dysregulated by HIV-1/METH co-treatment. Astrocytes were treated with or without METH (50 nM) and/or transduced with pseudotyped HIV-1 (500 RT) for 7 d prior to Seahorse Mito Stress test for metabolic assessment. Representative (A) OCR profile tracings from a representative astrocyte donor were graphed over time. Compiled data from seven separate biological donors quantifying fold changes in (B) basal respiration, (C) maximal respiration, (D) spare respiratory capacity, (E) ATP production, (F) proton leak and (G) non-mitochondrial OCR were graphed for statistical comparisons. Statistical significance was determined via one-way ANOVA followed by Fisher’s LSD test for multiple comparisons. Each dot on bar graphs represents the averaged data from a minimum of six replicates from a single biological astrocyte donor (n). FCCP, carbonyl cyanide-4 (trifluoromethoxy) phenylhydrazone; HIV-1, human innumodeficiency virus type 1; METH, methamphetamine; OCR, oxygen consumption rate; Oligo, oligomycin; Rot/AA, rotenone/antimycin A.

Optimal mitochondrial function is required for astrocytes to provide essential metabolic and antioxidant support to neurons. To evaluate how HIV-1 infection with or without chronic METH exposure impacts astrocyte mitochondrial function, astrocytes were transduced with pseudotyped HIV-1 (500 RT) and/or treated with METH (50 nM) for seven days prior to Seahorse XF Cell Mito Stress Test Profile assessment (Figure 3). An oxygen consumption rate (OCR) line tracing from a representative astrocyte donor graphed over time demonstrates primary human astrocytes have an elevated OCR after exposure to METH (50 nM) and/or transduction with pseudotyped HIV-1 compared to untreated controls (Figure 3A). Analysis of mitochondrial OCR demonstrated METH exposure and HIV-1 transduction, alone, increased basal respiration (Figure 3B; p<0.05), maximal respiration (Figure 3C; p<0.05), spare respiratory capacity (Figure 3D; p<0.05), ATP-linked respiration (Figure 3E; p<0.05), proton leak (Figure 3F; p<0.001), and non-mitochondrial respiration (Figure 3G; p<0.05) coinciding with our previous report in [17]. Importantly, the combination of METH exposure with HIV-1 transduction dysregulated METH- and HIV-1- induced increases in astrocyte respiratory activities. While maximal respiration (Figure 3C; p<0.05), spare respiratory capacity (Figure 3D; p<0.05), and proton leak (Figure 3F; p<0.001) were all significantly elevated during HIV-1/METH combination paradigms compared to control levels, there was no significant changes in basal respiration, ATP production, or non-mitochondrial OCR. In fact, exposure with METH in combination with HIV-1 transduction significantly decreased basal respiration (Figure 3B; p<0.05), maximal respiration (Figure 3C; p<0.05), spare respiratory capacity (Figure 3D; p<0.05), ATP-linked respiration (Figure 3E; p<0.05), and non-mitochondrial respiration (Figure 3G; p<0.01) when compared to HIV-1 transduction alone. These findings suggest chronic METH may suppress astrocyte respiratory capacity during HIV-1 infection.

**Figure 4:**
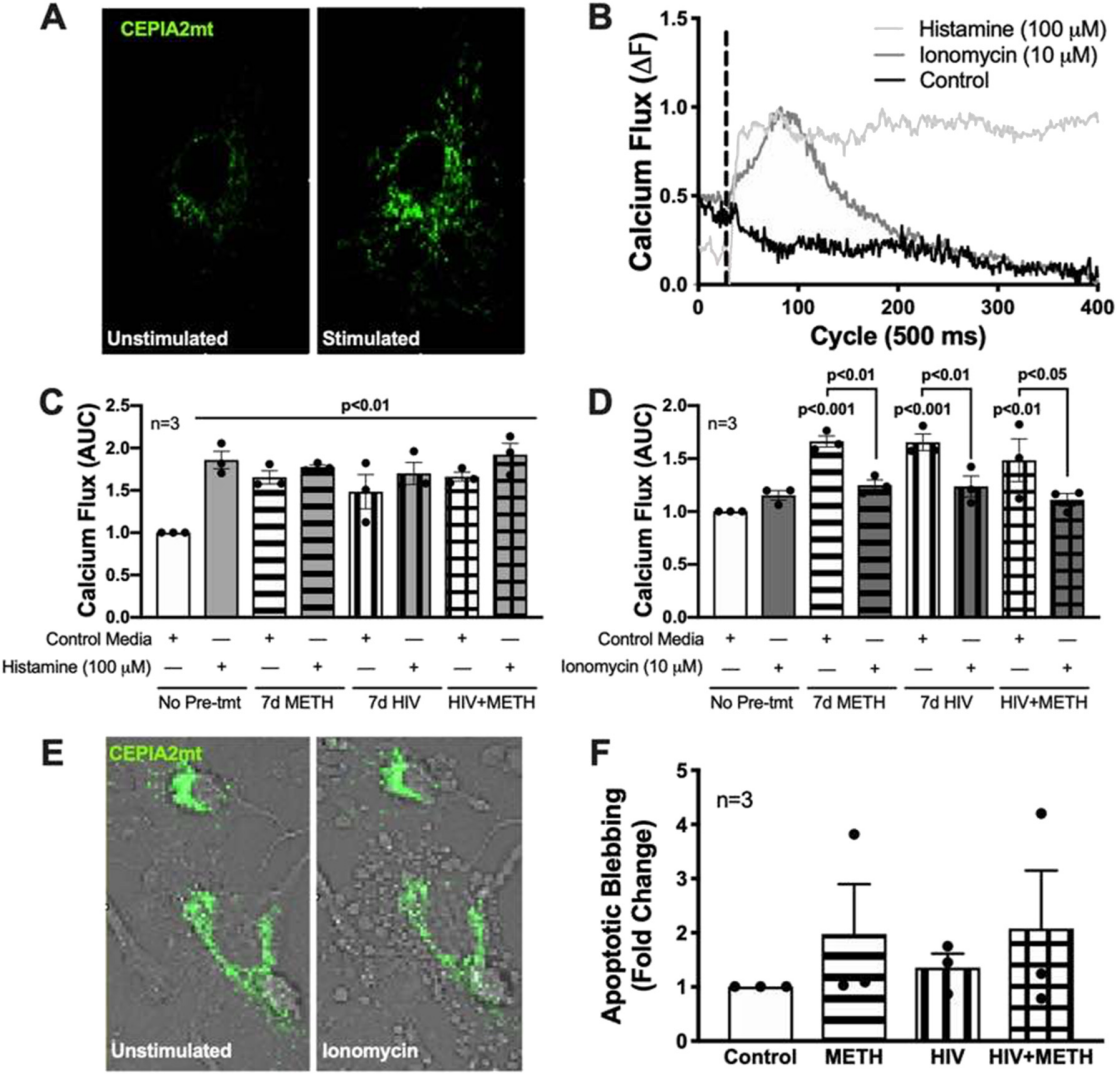
METH exposure and HIV-1 transduction alone or in combination increase basal mitochondrial calcium flux. Astrocytes were treated with or without METH (50 nM) and/or transduced with pseudotyped HIV-1 (500 RT) for 7 d prior to mitochondrial calcium imaging. Astrocytes were transfected with the calcium-measuring organelle-entrapped protein indicator targeted to the mitochondria and tagged with GFP (CEPIA2mt) reporter plasmid 48 h prior to calcium flux analysis. (A) Time series confocal imaging was used to measure changes in fluorescence following stimulation with media, histamine (100 μM), or ionomycin (10 μM). (B) Line tracings represents a single naïve astrocyte calcium flux response to acute media, histamine (100 μM), or ionomycin (10 μM) at 50 cycles (25 s). (C and D) Area under the curve (AUC) was calculated changes in fluorescence following treatment at 50 cycles (25 s) up to cycle 420 and graphed as fold changes to control. (E) Representative astrocyte showing apoptotic blebbing following chronic HIV/METH and acute ionomycin treatment. (F) Compiled quantification of astrocyte blebbing for 7 d pre-treatment conditions following acute ionomycin treatment. Individual dots represent compiled data from a minimum of 15 cells per biological astrocyte donor (n) graphed as fold changes. One-way ANOVA was performed for statistical analysis followed by Fisher’s LSD test for stand-alone comparisons to account for variation across different biological donors. AUC, area under the curve; CEPIA2mt, calcium-measuring organelle-entrapped protein indicator for the mitochondira; HIV-1, human immunodeficiency virus type 1; METH, methamphetamine.

Mitochondrial respiration is regulated by mitochondrial calcium uptake [40]. To measure changes in astrocyte mitochondrial calcium flux following chronic METH exposure and/or HIV-1 transduction, a genetically encoded calcium-measuring organelle-entrapped protein indicator was targeted to the mitochondria and tagged with GFP (CEPIA2mt) (Figure 4A). Calcium flux following stimulation with either control media, histamine (100 μM), or ionomycin (10 μM) were graphed over time (Figure 4B; Supplementary Figure 1). To illustrate differences in astrocyte mitochondrial calcium responses across acute treatments and chronic conditions, five individual cellular responses were graphed per condition from a representative donor (Supplementary Figure 1). Note, histamine triggers calcium release from the endoplasmic reticulum in a phospholipase C-dependent manner, where it can then be sequestered into mitochondria in an oscillatory mechanism via cationic exchange. Ionomycin is a potent calcium ionophore often used as a positive control to increase intracellular calcium mobilization and can trigger cell death through apoptosis. In control astrocytes (no pre-treatment; Figure 4B and Supplementary Figure 1A), histamine stimulation led to a sharp, robust, and prolonged increase in mitochondrial calcium flux while ionomycin led to a slower, lower, and shorter increase in mitochondrial calcium flux followed by a considerable decline below baseline. The area under the curve (AUC), following stimulation with either media, histamine (100 μM; Figure 4C), or ionomycin (10 μM; Figure 4D) was graphed for statistical comparisons. In control (no pretreatment) astrocytes, histamine induced a significant increase in mitochondrial calcium influx (p<0.01) (Figure 4C; light grey bars). However, astrocytes pretreated with 7 d METH and/or HIV-1 transduction no longer significantly respond to histamine. In fact, pretreated conditions stimulated with control media had significantly elevated basal mitochondrial calcium flux (p<0.01) compared to non-pretreated controls. The inability of astrocytes to respond to histamine following pretreatment with HIV-1 transduction and/or METH exposure suggests a dysregulated mitochondrial phenotype, where they are no longer able to internalize calcium or are at maximum capacity. Interestingly, astrocytes stimulated with ionomycin (Figure 4D; dark grey bars) following pretreatment with HIV-1 transduction and/or METH exposure, have a significant decrease in mitochondrial calcium load compared to their respective control conditions (p<0.05; clear bars). Decreases in calcium flux were accompanied by an increased visualization of apoptotic bleb formation (Figure 4E), suggesting a mitochondrial calcium-driven hypersensitivity to ionomycin-induced apoptosis. Quantification of apoptotic bleb formation trended with chronic METH paradigms; however, these findings were not statistically significant due to high donor variability (Figure 4F).

**Figure 5:**
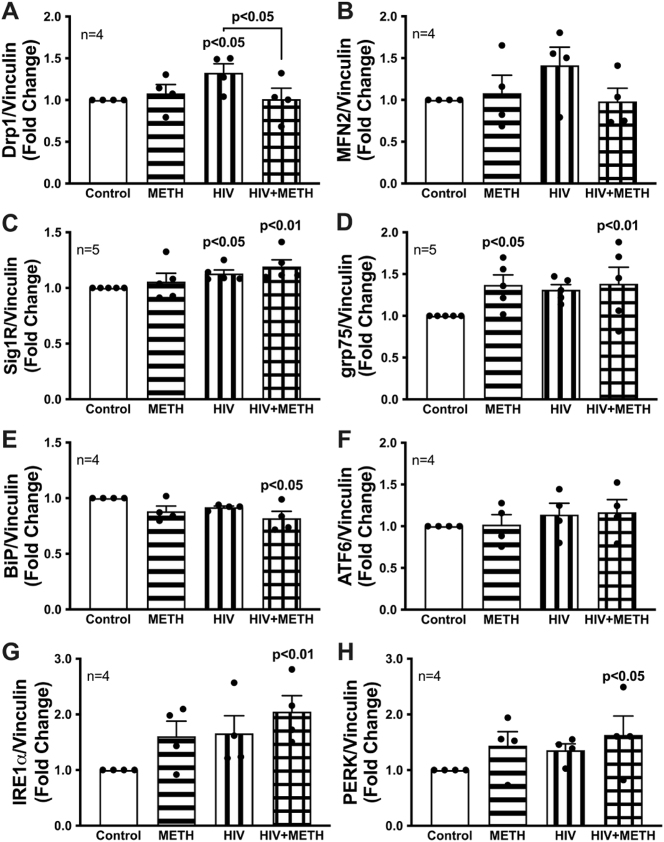
Chronic METH exposure and HIV-1 transduction alone or in combination alter UPR/MAM-associated protein expression in astrocytes. Astrocytes were treated with or without METH (50 nM) and/or transduced with pseudotyped HIV-1 (500 RT) for 7 d prior to lysate collection. Protein expression of (A) Drp1, (B) MFN2, (C) Sig1R, (D) grp75, (E) BiP, (F) ATF6, (G) IRE1α, and (H) PERK was measured via Simple Wes. Individual dots on bar graphs represent fold changes to for separate biological donors (n) normalized to vinculin as an internal control. Statistical significance was determined via one-way ANOVA followed by Fisher’s LSD test for multiple comparisons. ATF6, activation transcription factor 6; BiP, binding immunoglobin protein; Drp1, dynamin-related protein 1; grp75, glucose-regulated protein 75 kDa; HIV-1, human immunodeficiency virus type 1; IRE1α, inositol-requiring protein 1; METH, methamphetamine; MFN, mitofusin; PERK, protein kinase RNA-like endoplasmic reticulum kinase; Sig1R, sigma-1 receptor.

Contact and communication between the ER and mitochondria are essential for regulating mitochondrial dynamics, bioenergetics, and apoptotic signaling [16, 41]. Thus, changes in MAM-associated proteins were investigated in astrocytes following 7 d METH exposure and/or HIV-1 transduction using Simple Wes (Figure 5). Briefly, mitochondrial fission protein, Drp1, has been implicated as a potential target in HAND pathology [16]; however, these investigations were geared towards neurons and had contradictory findings. MFN2 is a mitochondrial fusion protein and has been highlighted as a key regulator of MAM tethering through interaction with UPR protein PERK; however, whether MFN2 positively or negatively regulates MAM tethering remains controversial [16]. Coinciding with a potential interplay in HAND, Drp1 levels were significantly upregulated following HIV-1 transduction (Figure 5A; p<0.05). Interestingly, when in combination with METH exposure, Drp1 levels were significantly decreased (p<0.05). MFN2 levels followed similar trends, although not significant (Figure 5B). Next, we evaluated expression changes of key proteins involved in MAM-mediated calcium transfer Sig1R (Figure 5C) and grp75 (*a.k.a.* mortalin; Figure 5D), both of which have been implicated as potential therapeutic targets for METH- or HIV-1- induced astrocyte dysfunction, respectively [20, 30]. HIV-1 transduction alone and in combination with METH significantly upregulated Sig1R expression (Figure 5C; p<0.05, p<0.01, respectively**)**. Similarly, grp75 expression was significantly upregulated following METH exposure alone (Figure 5D; p<0.05) and when in combination with HIV-1 transduction (p<0.01). Finally, the three UPR arms also arise as potential regulators of astrocyte-mediated neurotoxicity [10, 12, 25, 26, 28] and are now increasingly considered integral mediators within the MAM proteome beyond their classical UPR/ER stress functions [42, 43]. Following 7 d HIV-1 transduction combined with METH exposure, protein expression of the classical ER stress negative regulating binding partner, BiP, was significantly decreased (Figure 5E; p<0.05). Of the three UPR arms, ATF6 (Figure 5F), IRE1α (Figure 5G), and PERK (Figure 5H), HIV-1/METH in combination significantly increased expression of IRE1α (p<0.01) and PERK (p<0.05). Altogether, these findings demonstrate a disruption in astrocyte mitochondrial dynamic proteins with an augmented UPR/MAM mediator expression following HIV-1/METH co-treatment.

**Figure 6:**
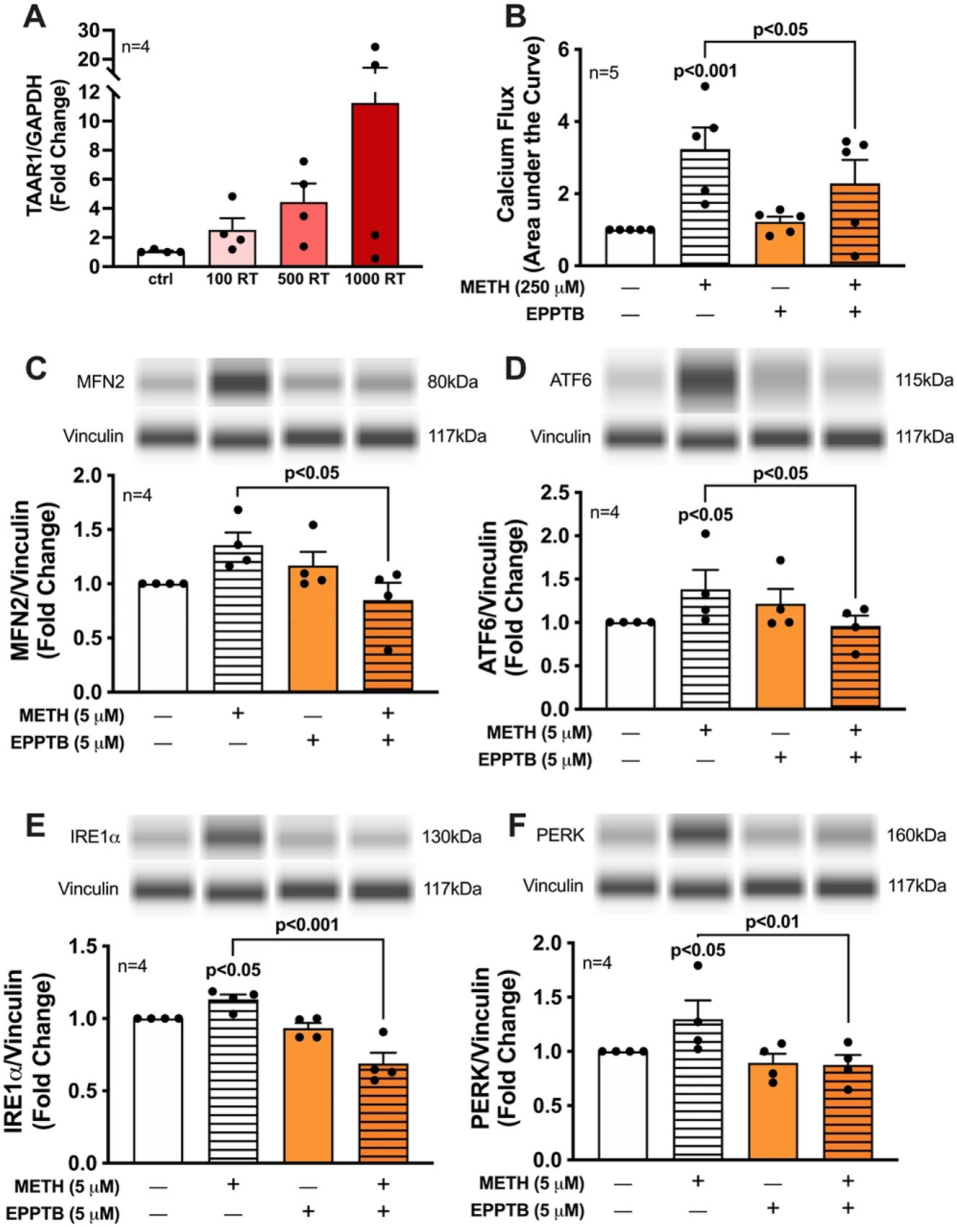
Astrocyte TAAR1 regulates METH-induced calcium flux and UPR/MAM mediator expression. (A) Astrocyte TAAR1 RNA expression was measured by RTPCR following transduction with pseudotyped HIV-1 (100–1000 RT) for 7 d. GAPDH was used as an internal control. Data from a representative donor is graphed. (B) For calcium flux analysis, naïve astrocytes were transfected with the cytosolic GFP-calmodulin calcium sensor (GCaMP6s) for 48 h prior to time series confocal imaging. Astrocyte cultures were exposed to EPPTB (5 μM), a TAAR1 selective antagonist, for 1 h prior to imaging. Area under the curve was calculated from changes in fluorescence following treatment with control media or METH (250 μM). Individual dots represent the average AUC from a minimum of 20 cells per biological donor and were graphed as fold changes. (C–F) astrocyte TAAR1 was inhibited by pretreatment with EPPTB (5 μM) for 1 h followed by acute METH (5 μM) stimulation for 8 h. protein expression of (C) MFN2, (D) ATF6, (E) IRE1α, and (F) PERK was measured via Simple Wes and normalized to vinculin as an internal control. Computer generated pseudo-blot bands were included above each panel to emphasize the effects of TAAR1 blockade using a representative donor. Individual dots on graphs represent fold changes for separate biological donors (n). Statistical significance was determined via one-way ANOVA followed by Fisher’s LSD test for stand-alone comparisons to account for variation across different biological donors. ATF6, activating transcription factor 6; EEPTB, N-(3-Ethoxy-phenyl)-4-pyrrolidin-1-yl-23-trifluoromethyl-benzamide; HIV-1, human immunodeficiency virus type 1; IRE1α, inositol-requiring protein 1α; METH, methamphetamine; MFN, mitofusin; PERK, protein kinase RNA-like endoplasmic reticulum kinase; TAAR1, trace amine-associated receptor 1.

The prospect of targeting ER stress and the three UPR arms to combat neurodegeneration has been widely explored as reviewed [44]. Indeed, our previous studies highlighted UPR arm, IRE1α, as a potential therapeutic target to combat astrocyte dysfunction during METH exposure or HIV-1 infection [17]. However, the multifunctional complexity of the distinct arms and their disparate induction may make them difficult targets for therapeutic application. Thus, identifying an upstream regulator of UPR/MAM dysfunction could provide a favorable avenue for therapeutic intervention. Our lab previously delineated TAAR1 as a novel METH-binding receptor in astrocytes and as an upstream regulator of astrocyte-mediated excitotoxicity during METH exposure [15, 29]. Additionally, TAAR1 expression is significantly elevated by HIV-1 and inflammation implicating a fundamental role for TAAR1 during HAND/METH comorbidity [15, 29]. Following the current model of pseudotyped HIV-1 infection in astrocytes, we confirmed a HIV-1 dose-dependent upregulation in TAAR1 mRNA expression (Figure 6A). To examine TAAR1 regulation on acute METH-induced intracellular calcium flux and UPR/MAM induction, astrocytes were pretreated with the selective TAAR1 antagonist, EPPTB (5 μM; orange bars) for 1 h prior to acute METH stimulation (horizontal lines) for cytosolic calcium signaling (250 μM; ∼5 min) (Figure 6B) or protein assessment (5 μM; 8 h) (Figure 6C–F). Changes in astrocyte intracellular calcium flux, was measured using a genetically modified GFP cytosolic calcium sensor (GCaMP6s) followed by time series confocal analysis (Figure 6B) [25, 45]. Acute stimulation of naïve astrocytes with METH (250 μM; ∼5 min) significantly increased intracellular calcium flux (p<0.001). Importantly, METH-induced calcium flux was suppressed when astrocyte TAAR1 was blocked with EPPTB (p<0.05). Similarly, METH-induced protein expression of MFN2 (Figure 6C; p<0.05), ATF6 (Figure 6D; p<0.05), IRE1α (Figure 6E; p<0.001), and PERK (Figure 6F; p<0.01) were also significantly suppressed by EPPTB pretreatment compared to METH treatment alone (5 μM; 8 h). Together these data implicate astrocyte TAAR1 as an upstream regulator and potential therapeutic target for METH-induced UPR/MAM stress responses.

## Discussion

**Figure 7:**
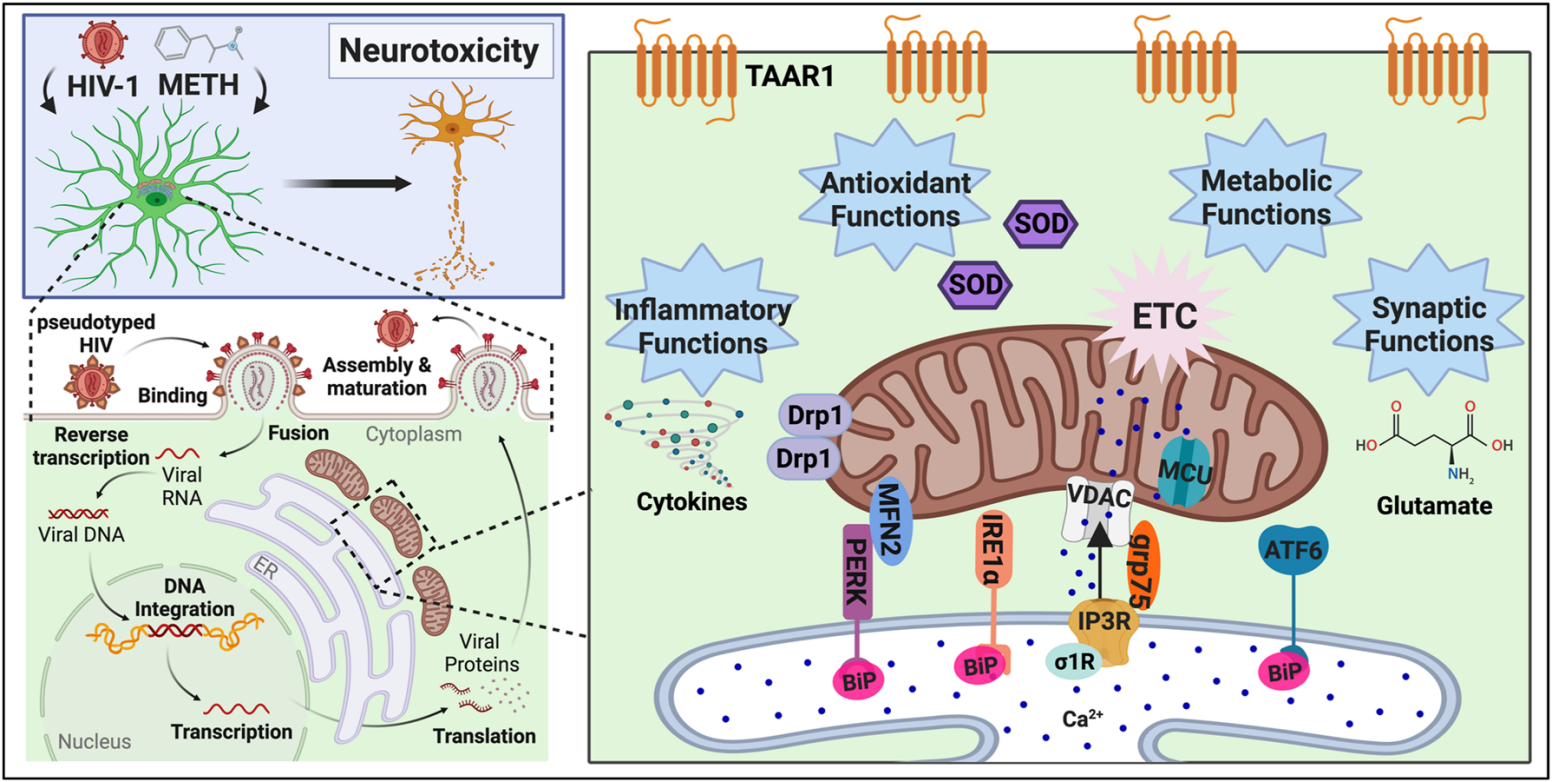
The ER-mitochondrial interface in astrocytes during METH exposure and HIV-1 infection. Following METH exposure and/or pseudotyped HIV-1 transduction, astrocytes shift their physiology and function, which can impair astrocyte-mediated neuroprotection and increase neurotoxicity. The ER-mitochondrial interface arises as a key pathological platform for HIV-1/METH-mediated astrocyte dysfunction. Notably, astrocyte HIV-1 transduction was increased by chronic METH exposure. The effects of HIV-1 transduction and METH exposure, alone or in combination, dysregulate astrocyte calcium homeostasis, mitochondrial function and dynamics, and UPR/MAM protein expression. These changes in ER-mitochondrial function and physiology are important regulators for astrocyte inflammatory, antioxidant, metabolic, and synaptic functions. Image made with BioRender. ATF6, activating transcription factor 6; BiP, binding immunoglobin protein; calcium, Ca^2+^; Drp1, dynamin-related protein 1; ETC, electron transport chain; grp75, glucose-regulared protein 75 kDa; HIV-1, human immunodeficiency virus type 1; IP_3_R, inositol 1,4,5,-triphosphate receptors; IRE1α, inositol-requiring protein 1α; MCU, mitochondrial calcium uniporter; METH, methamphetamine; MFN, mitofusin; PERK, protein kinase RNA-like endoplasmic reticulum kinase; σ1R, sigma-1 receptor; SOD1, superoxide dismutase 1; TAAR1, trace amine-associated receptor 1.

The present investigations evaluated the etiology of HAND and METH use disorders in respect to key neurosupportive glial cells, astrocytes. During a neuropathological challenge, such as HIV-1 infection of METH exposure, ‘activation’ of astrocytes can shift their primary functions. Acutely, these functional shifts can optimize neuronal protection, such as a glial scar formation during a stroke. However, failure to restore homeostatic balance, leads to chronic states of astrocyte activation, like that in HAND or METH use disorders, that can inadvertently lead to neuronal toxicity. Here, we interrogate the effects of HIV-1/METH on astrocyte infection, inflammation, mitochondrial function and calcium homeostasis, and UPR/MAM protein expression. These results are summarized in Table 2. Moreover, Figure 7 illustrates the current findings in scope with some of our previous works [17].

**Table 2:** Summary of HIV-1/METH co-treatment on astrocyte HIV-1 transduction and physiology. Arrows represent directional trends (up or down) for each respective condition/comparison across different experiments (–, N/A or not significant). Comparisons are relative to control astrocytes, unless otherwise noted (i.e. HIV-1/METH vs. HIV-1 alone).

Experiment	METH alone	HIV-1 alone	HIV-1/METH combined	HIV-1/METH vs. HIV-1 alone
HIV-1 DNA integration	–	↑	↑↑	↑
HIV-1 protein expression	–	↑	↑↑	↑
HIV-1 viral production	–	↑	↑↑	↑
Inflammation	–	–	–	–
Antioxidant expression	↓	↓	↓↓	–
Mitochondrial respiration	↑	↑↑	↑	↓
Mitochondrial calcium flux	↑	↑	↑	–
Mitochondrial dynamic protein expression	–	↑	–	↓
MAM calcium regulator expression	↑	↑	↑↑	–
UPR protein expression	↑	↑	↑↑	–

Low-dose chronic METH exposure enhanced HIV-1 infection via increased HIV-1 DNA integration, HIV-1 protein expression, and extracellular viral production with no impact on astrocyte inflammatory profile (Figure 2). As previously reported in Proulx et al. 2022, chronic METH exposure or HIV-1 transduction alone elevates astrocyte mitochondrial respiration [17]. Here, co-treatment of HIV-1/METH appeared to suppress astrocyte basal, maximal, and spare respiratory capacity compared to HIV-1 transduction alone (Figure 3). Likewise, HIV-1-induced expression of mitochondrial fission (Drp1) and fusion (MFN2) proteins were similarly downregulated following combination with chronic METH exposure (Figure 5). It is important to note that changes in mitochondrial respiration and dynamics are not a fully inclusive reflection of astrocyte mitochondrial health. For instance, changes in mitochondrial respiration and dynamics may not translate to neuroprotective outcomes (*i.e.*, production and release of metabolites and antioxidants). Coupling these data with decreased antioxidant SOD1 levels (Figure 2), suppressed ATP production and persistent proton leak (Figure 3), and increased basal mitochondrial calcium load (Figure 4), rather supports dysfunctional astrocyte mitochondria during HIV-1/METH in combination. Additional studies are needed to examine shifts in metabolite provision and/or production of toxic radicals. Moreover, as both a mitochondrial fission and fusion proteins are dysregulated by METH and/or HIV-1, further examination into the structural consequences on astrocyte mitochondria morphology are essential to better understand the effects of HIV/METH on astrocyte mitochondrial biology.

Mitochondrial bioenergetics, dynamics, and integrity are regulated by the MAM interface. ER-associated messengers, including calcium and the UPR sensors, are highlighted as potential therapeutic targets to combat mitochondrial dysfunction [16, 17]. Here, HIV-1/METH co-exposure significantly increased two key regulators of MAM-mediated calcium transfer, grp75 and Sig1R, and UPR protein expression of IRE1α and PERK (Figure 5). Notably, protein expression of the UPR stress sensor and negative regulator, BiP, was significantly decreased following HIV-1/METH co-treatment. BiP binds to the three UPR arms as a mechanism to inhibit ER stress. Dissociation of BiP from the UPR arms is the first step of ER stress signaling cascade. Decreased abundance of BiP in astrocytes following HIV-1/METH co-treatment could indicate a deficiency in misfolded protein chaperones and/or unchecked regulation of ER stress signaling. Moreover, the relative expression of the three UPR arms compared to BiP abundance could have further implications of the state of ER stress and/or homeostasis. Another important consideration is that the three arms of the UPR have non-canonical functions beyond their classical ER stress cascades. Thus, increased protein expressions of PERK and IRE1α could be independent of their conventional UPR functions.

Briefly, cytosolic grp75 stabilizes the association of inositol 1,4,5-triphosphate receptors (IP_3_R; ER membrane) and voltage-dependent anion-selective channel (VDAC; outer mitochondrial membrane) to facilitate ER-mitochondria calcium transfer while Sig1R promotes calcium transfer by associating with IP_3_R [46]. IRE1α has recently been implicated in ER-mitochondrial calcium transfer, mitochondrial respiration and redox homeostasis through associations with IP_3_R [47, 48] and/or Sig1R [49]. Meanwhile, PERK is proposed as a key regulator of MAM tethering through direct interaction with MFN2 and is also linked to regulating mitochondrial dynamics and bioenergetics [23, 24, 50]. It is important to note that as part of their classical ER stress signaling cascades, both PERK and IRE1α are phosphorylated, which was not investigated in this report. Additional studies are needed to decipher the canonical versus non-canonical consequences of PERK and IRE1α upregulation in astrocytes following HIV-1/METH co-treatment.

Two METH-binding receptors have been identified in astrocytes, Sig1R and TAAR1, both of which are upregulated by HIV-1, indicating that astrocytes may be especially sensitive to HIV-1/METH comorbidity. TAAR1 regulates excitatory amino acid transporter 2 expression and glutamate uptake during HIV-1 and/or METH treatment, highlighting TAAR1 as a novel regulator of astrocyte-mediated excitotoxicity [15, 29]. We evaluated TAAR1 as a target for METH-mediated calcium dysregulation and UPR/MAM induction. Indeed, selective inhibition of TAAR1 significantly blocked acute METH-induced intracellular calcium flux and UPR/MAM protein expression, including IRE1α (Figure 6). Thus, targeting TAAR1 could not only modulate astrocyte IRE1α signaling but could also control other key ER/UPR/MAM messengers (PERK, ATF6, MFN2, and calcium). Altogether, these findings demonstrate HIV-1/METH-induced ER-mitochondrial dysfunction in astrocytes, and astrocyte TAAR1 may be an upstream regulator for HIV-1/METH-mediated UPR/MAM dysfunction. The potential therapeutic targeting of TAAR1 to modulate ER-associated signaling pathways, including UPR and calcium, may provide a novel mechanism to combat astrocyte dysfunction during HIV-1/METH neuropathology. Additional studies are needed to evaluate the role of TAAR1 on astrocyte ER-mitochondrial function and physiology, including the interplay of mitochondrial dynamics (Drp1 and MFN2) and MAM-mediated calcium transfer (grp75 and Sig1R).

For example, decreased expression of Drp1 has previously coincided with increased HIV-associated neurocognitive decline in HIV+ brain tissues and astrocyte-restricted HIV-1 gp120 transgenic mice [51]. Overexpressing Drp1 reversed gp120-meditated neuronal dysfunction reducing both neuroinflammation and neurodegeneration. Decreased Drp1 and MFN2 expression has also been evident in T cells following exposure to HIV-1 Vpr protein. These changes coincided with impaired ER-mitochondrial interaction and morphology and induced mitochondrial depolarization and deformation. Overexpressing MFN2 or Drp1 was able to prevent T cell mitochondrial depolarization and deformation [52]. Thus, the role and potential therapeutic targeting of MFN2 or Drp1 to promote astrocyte neuroprotective phenotypes and combat astrocyte-associated neurotoxic consequences requires additional investigations.

Furthermore, Sig1R was identified as a key modulator in HIV-1/METH pathobiology in CD4+ T cell activation and infection [53]. Targeting Sig1R has been proposed to combat both MAM dysfunction and neuroinflammation in neurological diseases [54–56]; however, these studies are predominately in neurons or whole brain tissues. In previous works, overexpression of grp75 in astrocytes expressing HIV-1 Tat was able to prevent astrocyte mitochondrial dysfunction and fragmentation and protect neurons from astrocyte-mediated neurotoxicity by reducing the release of excess ATP, inflammatory cytokines, and extracellular glutamate [20]. Moreover, blocking ER-mitochondrial calcium transfer via VDAC [19] or mitochondrial calcium uniporter on the inner mitochondrial membrane [10] restores astrocyte neurotrophic phenotypes. These studies emphasize the therapeutic application of MAM-mediated calcium transfer to combat astrocyte-mediated neurotoxicity during HAND/METH.

While some studies of HIV-1 in astrocytes have danced around the idea of ER-mitochondrial interplay through calcium signaling [25, 57, 58], no research to date has investigated MAMs as potential pathological platforms for HAND or METH pathogenesis. In fact, the only evidence of MAMs in HIV-1 biology was reported in T cells showing that HIV-1 Vpr localizes to both the ER and mitochondria, and MAMs serve as a possible route for intracellular trafficking of Vpr [52]. Moreover, there are only two previous investigations of MAMs in astrocytes, which were focused on astrocyte-mediated vascular remodeling [59] and synaptic homeostasis [60]. More studies are needed to elucidate the roles of MAM mediators and mechanisms in astrocyte biology and HIV-1/METH pathogenesis, especially if MAMs may be potential therapeutic targets to regulate astrocyte-mediated neuroprotection.

Ultimately, HIV-1 is a lifelong, chronic disease that increases the risks for an array of comorbidities including neurological impairment and substance use. People living with HAND or METH use disorders can have widespread and long-lasting psychological and neurophysiological consequences. Importantly, substance use disorders, including METH, are disproportionately elevated among PLWH and influence both the development and severity of HAND, emphasizing the need to better understand the cellular complexities of these comorbidities. While these studies identified several potential targets that may help ameliorate HIV-1 and/or METH disease outcomes, they can only treat the pathophysiological mechanisms at play in astrocytes. Additional studies will need to validate the potential to optimize the beneficial coupling between astrocytes and neurons. In the absence of a cure for HIV-1 and addiction, developing therapies to regulate astrocyte functional responses to chronic disease may ultimately preserve neuronal function and improve outcomes in various neurological disorders.

## Supplementary Material

Supplementary Material Details
