# Supplementary material for: HIV-1 and methamphetamine co-treatment in primary human astrocytes: TAARgeting ER/UPR dysfunction
Source: NeuroImmune Pharm Ther. 2024 Feb 19;3(2):139–54. doi: 10.1515/nipt-2023-0020 (PMC11338011; doi:10.1515/nipt-2023-0020)
Supplement: Supplementary file 1 — Supplementary Material Details [file j_nipt-2023-0020_suppl_001.docx]

Supplemental Figures


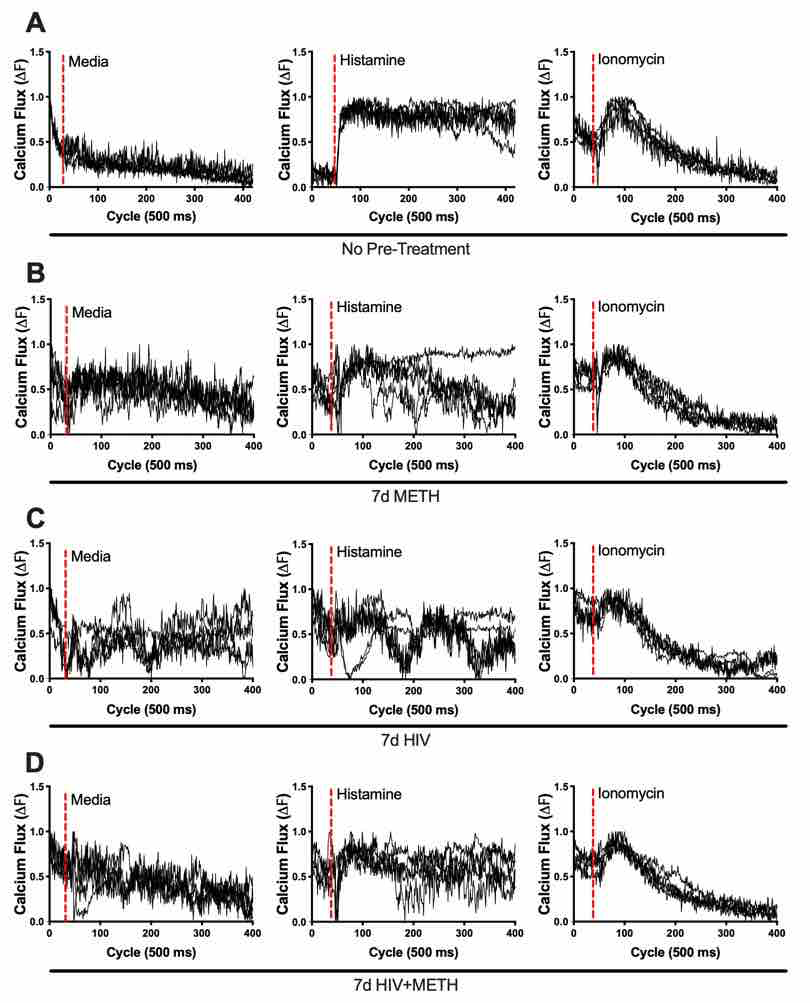


**Supplementary Figure 1:** **Representative tracings of astrocyte mitochondrial calcium flux in response to acute histamine or ionomycin following 7 d METH exposure and/or HIV-1 transduction.** (A) Naïve astrocytes or astrocytes treated for 7 d with (B) METH (50 nM) (C) pseudotyped HIV-1 (500 RT) or (D) HIV/METH in combination were transfected with the calcium-measuring organelle-entrapped protein indicator targeted to the mitochondria and tagged with GFP (CEPIA2mt) reporter plasmid 48 h prior to calcium imaging. Time series confocal imaging was used to measure changes in fluorescence following stimulation with media, histamine (100 μM), or ionomycin (10 μM) at 50 cycles (25 sec) up to cycle 420 (~ 4 min). Graphs illustrate line tracings from five representative astrocyte calcium flux responses. Calcium flux was calculated by: ΔF=(F−Fmin)/(Fmax−Fmin) and graphed over time, where F is the fluorescence intensity at any given time; Fmin is the minimum fluorescence intensity, and Fmax is the maximum fluorescence intensity.


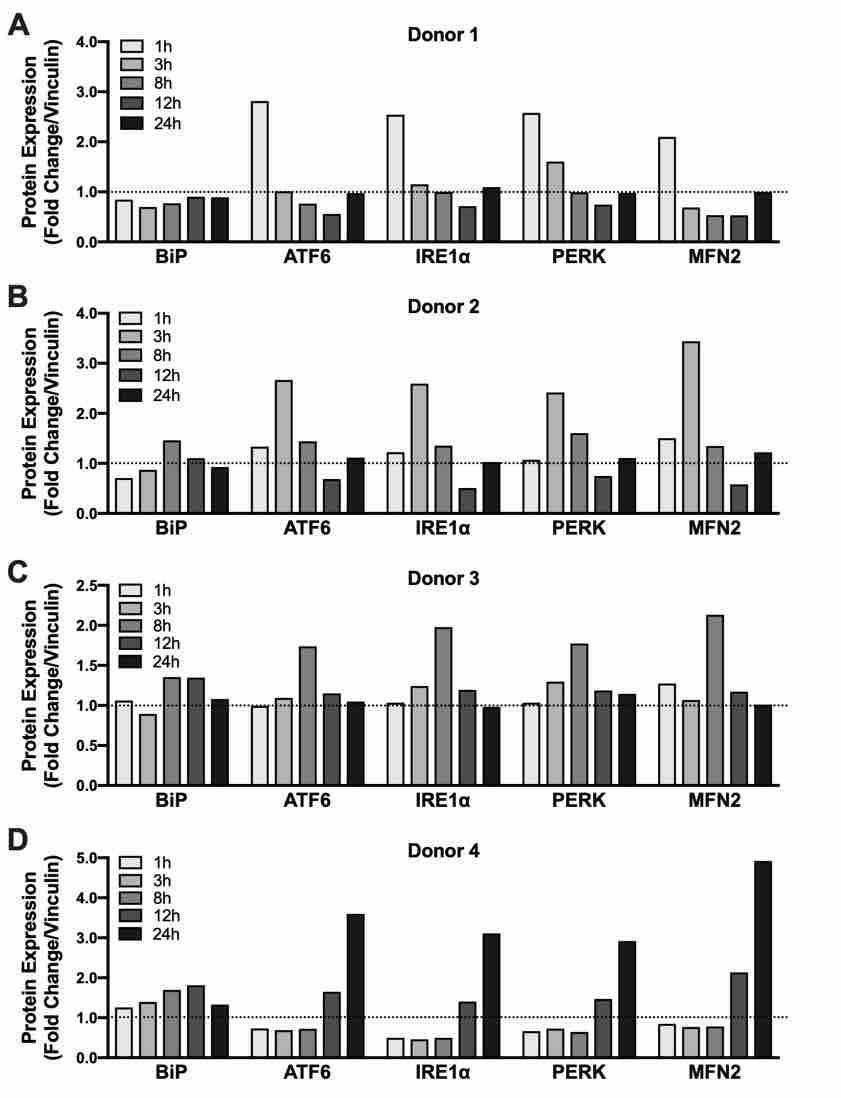


**Supplementary Figure 2:** **Acute METH exposure induces unique UPR/MAM protein induction across different primary human astrocyte cultures.** Astrocytes from four donor cultures (A-D) were treated with acute METH (5 μM) for 1, 3, 8, 12, and 24 h (shading darkens with time). Protein expression of BiP, ATF6, IRE1α, PERK, and MFN2 was measured *via* Simple Wes and graphed as fold change to untreated controls to illustrate trends across separate biological donors. Vinculin was used as an internal control.

**Materials and Methods**

*Primary human astrocyte cultures*: Astrocytes were plated in T25 or T75 flasks at 3.33 or 10 million cells, respectively, or in 6-well plates at 2 million cells per well and allowed to adhere overnight, prior to treatment. Chronically treated astrocytes were passaged and replated at day 5 post treatment (48 h prior experimental assessment) for subsequent studies.

*METH and EPPTB treatment*: Astrocytes were treated with METH (cat # M8750, Sigma-Aldrich, St. Louis, MO, USA) for chronic experiments (50 nM; 7 d) and acute protein expression (5 µM; 8 h) or calcium signaling (250 µM; 5 min). Dose and time kinetics were determined based on the prolonged METH ranges (60 – 600 nM) and physiological peak (6 μM – 2 mM) found *in vivo* and our previous investigations [[7](#_ENREF_7), [11](#_ENREF_11), [13](#_ENREF_13), [17](#_ENREF_17), [27](#_ENREF_27), [29](#_ENREF_29)]. Thus, higher doses (5 – 250 μM) were used for acute peak assessment and lower doses (50 nM – 250 nM) were used for chronic studies to model the low levels of residual METH present in the brains of chronic METH users in between binges. To inhibit METH-binding receptor, TAAR1, astrocytes were treated with N-(3-Ethoxy-phenyl)-4-pyrrolidin-1-yl-23-trifluoromethyl-benzamide (EPPTB, 5 μM, cat# 4518 Tocris-BioTechne, Minneapolis, MN) for 1 h prior to acute METH treatment. EPPTB is a cell permeable selective antagonist for TAAR1 [[15](#_ENREF_15), [61](#_ENREF_61)]

*Pseudotyped HIV-1*: A pseudotyped HIV-1 that modifies the viral coat with vesicular stomatitis virus glycoprotein (VSVg) to permit entry independent of CD4 expression was constructed and characterized as previous described [[17](#_ENREF_17)]. Briefly, human embryonic kidney (HEK) 293 T cells were co-transfected with HIV-1 infectious molecular clone (pNL4-3; cat # ARP-114, NIH HIV Reagent Program, Manassas, VA, USA, contributed by Dr. M. Martin) and p-human elongation-factor (pHEF)-VSVg (plasmid # 22501, Addgene, Watertown, MA, USA, a gift from Dr. Sergey Kasparov) by calcium phosphate precipitation per CalPhos Mammalian Transfection kit instructions (Clontech Laboratories, Inc., Mountain View, CA, USA) [[14](#_ENREF_14), [17](#_ENREF_17)]. The cells were incubated overnight, washed three times with PBS, and fresh culture media was added. Supernatants were collected 48 h post-wash, and progeny pseudotyped HIV-1 was quantified by reverse transcriptase (RT) activity *via* radiometric RT assay [[14](#_ENREF_14)]. After RT quantification, astrocytes were transduced with pseudotyped HIV-1 (500 RT) and remained in culture for a total of 7 d prior to experimental analyses. On day one post-transduction, cells were washed three times with PBS and fresh media was added for the remaining days in culture. Confirmation and characterization of infection in primary human astrocytes was previously described [[17](#_ENREF_17)] based on HIV-1 DNA integration, detectable levels of viral protein expression, and minimal cytotoxicity to best mimic chronic *in vivo* HIV-1 astrocyte infection.

*Reverse transcriptase (RT) activity*: Supernatants from primary human astrocyte cultures were collected 7 d post-transduction from the same wells used for protein assessment. Reverse transcriptase activity in supernatants was quantified as a measure of viral particle production following HIV-1 transduction. Triplicate aliquots of 10 μl per viral sample were lysed with equal volume of dissociation buffer (100 mM Tris-HCl [pH 7.9], 300 mM KCl, 10 mM dithiothreitol [DTT], 0.1% NP-40) in a round-bottom-well plate and incubated at 37°C and 5% CO2 for 15 min. The level of reverse transcriptase was measured in an enzymatic assay by adding 25 μl reaction buffer [50 mM Tris-HCl (pH 7.9), 150 mM KCl, 5 mM DTT, 0.05% NP-40, 15 mM MgCl2, 5 μl of 1 mg/ml poly(A)·(dT)15 (Roche, Indianapolis, IN), and 1 μCi/ml of [3H]dTTP tetrasodium salt (PerkinElmer, Inc., Waltham, MA)] Reactions incubated for 18 to 24 h at 37°C and 5% CO2. The reverse-transcribed cDNA was precipitated with 50 μl of ice-cold 10% trichloroacetic acid (TCA) for fifteen minutes before harvesting each sample. Precipitated DNA was transferred to a filter plate (MultiScreen 96-well harvest plates, catalog no. MAHFB1H60; Millipore, Burlington, MA) using a FilterMate harvester (PerkinElmer, Inc.), washed three times with 5% TCA and dehydrated with 95% ethanol. A MicroBeta2 scintillation counter (PerkinElmer, Inc.) was used to detect the level of 3H incorporation to quantify reverse transcriptase activity as a measure of viral concentration.

*HIV-1 DNA integration assay*: Astrocytes plated in 6-well plates at 1 million cells per well were collected using 0.05% Trypsin-EDTA (cat # T3924; Sigma-Aldrich) 7 d post treatment with or without METH (50 nM) and/or pseudotyped HIV-1 (500 RT), as described above. Astrocytes were washed with PBS prior to collection and subsequent DNA isolation using QIAamp DNA Micro Kit per manufacturer’s instructions (Qiagen, Germany). Integration of HIV-1 into the human genome was confirmed using a nested, two-step PCR HIV-1 integration assay followed by visualization of final PCR product *via* electrophoresis as previously described [[14](#_ENREF_14), [17](#_ENREF_17)]. Briefly, the first step uses sequence-specific primers for Alu (a highly repetitive short DNA segment in the human genome) and HIV-1 gag (HIV-1 gene encoding the main structures of the virion particle) to amplify HIV-1 DNA integrated into the human host genome. The second PCR product is specific for R/U5 DNA regions within the HIV-1 long terminal repeat. A HIV-1 lymphadenopathy-associated virus-infected 8E5 cells (8E5, cat # ARP-95, NIH HIV Reagent Program, contributed by Dr. Thomas Folks) were used as a positive control.

*Protein expression via Simple Wes*: Astrocytes treated with or without METH (50 nM) and/or pseudotyped HIV-1 (500 RT) were plated in 6-well plates at 2 million cells per well. Lysates were collected 7 d post treatment using mammalian protein extraction buffer (MPER, cat # PI78505, Thermo Fisher Scientific) with 1X protease and phosphatase inhibitors (cat # P8340, cat # P0044 and cat # P2850, Sigma-Aldrich). Protein concentrations were quantified *via* bicinchoninic acid (BCA) assay per manufacturer’s instructions (cat # 23225, Thermo Fisher Scientific). Lysates were then diluted in 0.1X Wes Sample Buffer to 2 μg/μL for HIV-1 protein assessment or 0.5 μg/μL for all other targets. Protein expression was determined using Simple Wes automated capillary system with 12 – 230 kDa separation modules per manufacturer’s instructions and as previously described (cat # SM-W004, ProteinSimple)[[14](#_ENREF_14), [17](#_ENREF_17)]. All reagents except for primary antibodies were provided in separation and detection modules from ProteinSimple. Dilutions for primary antibodies were standardized for each target. Targets included: ATF6 (clone D4Z8V, cat # 65880, Cell Signaling Technology; 1:50, ~115 kDa), binding immunoglobulin protein (BiP; clone C50B12, cat # 3177, Cell Signaling Technology, Danvers, MA, USA; 1:250, ~71 kDa), Drp1 (clone 4E11B11, cat # 14647, Cell Signaling Technology; 1:50, ~85 kDa), grp75 (cat # sc-133137, Santa Cruz Biotechnology; 1:50, ~67 kDa), HIV-1 negative factor protein (Nef; cat # ARP-3689, HIV Reagent Program, contributed by Dr. James Hoxie; 1:25, ~30 kDa), HIV-1 capsid protein (p24; cat # ab43037, Abcam, Cambridge, UK; 1:25, ~32 kDa), IRE1α (clone 14C10, cat # 3294, Cell Signaling Technology; 1:50, ~130 kDa), PERK (clone D11A8, cat # 5683, Cell Signaling Technology; 1:50, 170 kDa), SOD1 (cat # AF3418, Novus Biologicals; 1:200, 20 kDa), Sig1R (cat # NBP1-82479, Novus Biologicals; 1:50, 29 kDa), and vinculin (clone E1E9V, cat # 13901, Cell Signaling Technology; 1:20,000, ~117 kDa). Vinculin was used as internal control for protein normalization. Data and digitalized blot images were generated using Compass for SW software (Version 4.0.0). Note: Molecular weights can shift in Simple Wes compared to classical western blot.

*Chemokine expression*: Supernatants from primary human astrocyte cultures were collected 7 d post-transduction from the same wells used for protein assessment. Colorimetric enzyme-linked immunosorbent assays (ELISA) were performed according to the manufacturer’s instructions to quantify C-C motif chemokine ligand 2 (CCL2) and C-X-C motif chemokine ligand 8 (CXCL8) secretion levels in culture supernatants (CCL2, cat # DCP00; CXCL8, cat # D8000C, R&D Systems) Protocol was standardized for primary human astrocytes as previously described [[14](#_ENREF_14), [32](#_ENREF_32)].

*Mitochondria bioenergetics*: Metabolic profiles were performed using Seahorse XFe96 analyzer technology per Seahorse XF Cell Mito Stress Test Kit User Guide instructions and as previously described (cat # 103015-100, Agilent Technologies, Santa Clara, CA, USA) [[17](#_ENREF_17), [62](#_ENREF_62)]. Briefly, astrocytes treated with or without METH (50 nM) and/or pseudotyped HIV-1 (500 RT) for a *total* of 7 d were plated 48 h prior to Mito Stress Test in Seahorse 96-well plates at 25,000 – 30,000 cells per well. There was a minimum of 6 wells per condition for each biological donor. Approximately 1 h before experiment, astrocytes were washed, and media was exchanged with seahorse assay media. The protocol for Seahorse XF Cell Mito Stress Test was carried out with injections of oligomycin (Oligo; 2 µM), carbonyl cyanide-4 (trifluoromethoxy) phenylhydrazone (FCCP; 2 µM), and rotenone/antimycin A (Rot/AA; 0.5 µM) to modulate components of the electron transport chain. These treatments allow measurement of key parameters of mitochondrial function. Data were collected using Wave V2.6.1.56 software and exported with Seahorse XF Cell Mito Stress Test Report Generator.

*Mitochondrial calcium signaling:* A green, fluorescent calcium-measuring organelle-entrapped protein indicator for the mitochondria (pCMV-CEPIA2mt, plasmid # 58218, Addgene) was a gift from Masamitsu Iino [[63](#_ENREF_63)]. Chronic HIV-1 infected and/or METH treated astrocytes were transfected using the Amaxa P3 primary cell 96-well kit, nucleofector and shuttle attachment (cat # V4SP-3960, Lonza, Walkersville, MD, USA) per manufacturer’s instructions with modification as previously published [[25](#_ENREF_25)]. Briefly, 500 ng of plasmid DNA was transfected per 1.6 million astrocytes in 20 µL of nucleofection reagent. Astrocytes were plated at 150,000 cells per well in tissue culture treated, six channel μ-slides (0.4 VI, cat # 80606, ibidi, Madison, WI, USA) and allowed to recover for 48 h before downstream assessments. Each condition was performed in duplicate wells for each biological donor with a minimum of 15 cells imaged per donor. Changes in calcium flux were imaged with a 40x objective at excitation of 450 – 490 nm and emission of 593 – 668 nm, using time series confocal microscopy *via* Carl Zeiss LSM 510 (Jena, Germany) [[25](#_ENREF_25)]. Time-lapse micrographs were acquired every 500 msec for 4 min (480 cycles). Astrocytes were treated with control media, METH (250 μg/ml), histamine (100 μM), or ionomycin (10 μM) at 25 s (50 cycles). Analysis was performed using Fiji ImageJ software (Version: 2.0.0-rc-69/1.52i; National Institutes of Health, Bethesda, MD, USA). Individual cells were outlined and the change in fluorescence was calculated by: ΔF=(F−Fmin)/(Fmax−Fmin), where F is the fluorescence intensity at any given time; Fmin is the minimum fluorescence intensity recorded, and Fmax is the maximum fluorescence recorded. Calcium flux line tracings illustrate the ΔF at any given time point. Area under the curve (AUC) was calculated by the sum of ΔF following acute treatment (cycles 50-420).

*Cytosolic calcium signaling*: A circularly permutated green fluorescent protein (GFP) with a calmodulin tag was constructed as an ultrasensitive calcium sensor (GCaMP6s) and gifted from Dr. Douglas Kim (pGP-CMV-GCaMP6s, plasmid # 40753, Addgene) [[45](#_ENREF_45)]. Astrocytes were transfected with GCaMP6s using same nucleofection method as above. Briefly, 250 ng of plasmid DNA was transfected per 1.6 million astrocytes in 20 µL of nucleofection reagent. Astrocytes were plated at 100,000 cells per well in tissue culture treated, six channel μ-slides (0.4 VI, cat # 80606, ibidi, Madison, WI, USA) and allowed to recover for 48 h before downstream assessments. Each condition was performed in duplicate wells for each biological donor with a minimum of 20 cells imaged per donor. Changes in calcium flux were imaged with a 20x objective at excitation of 450 – 490 nm and emission of 593 – 668 nm, using time series confocal microscopy *via* Carl Zeiss LSM 510 (Jena, Germany) [[25](#_ENREF_25)]. Time-lapse micrographs were acquired every 500 msec for 5 min. Astrocytes were treated with control media or METH (250 μg/ml) at 10 s (20 cycles) then ionomycin (10 μM) at 225 s (450 cycles). Analysis was performed using Fiji ImageJ software (Version: 2.0.0-rc-69/1.52i; National Institutes of Health, Bethesda, MD, USA). Individual cells were outlined and the change in fluorescence was calculated by: ΔF=(F−F0)/(Fmax−F0), where F is the fluorescence intensity at any given time; F0 is the baseline (1 – 20 cycles) fluorescence intensity, and Fmax is the maximum fluorescence intensity when exposed to ionomycin (450 – 600 cycles). Calcium flux line tracings illustrate the ΔF at any given time point. Area under the curve (AUC) was calculated by the sum of ΔF between METH/media and ionomycin treatment (cycles 20 – 450).

*Data analysis and interpretation*: All experiments were performed in at least three separate biological donors. Data in graphs were analyzed and presented using GraphPad Prism (version 8.1.1, GraphPad Software, San Diego, CA, USA) as mean +/- standard error of the mean with replicates from each donor compiled and represented as a single dot on graphs. Paired two-tailed t-tests were performed for HIV-1 transduction data to compare HIV-1 alone versus HIV-1/METH (Figure 1A-B). All other statistics were determined *via* one-way ANOVA followed by Fisher’s LSD test for multiple comparisons to account for variability across different biological donors. Significance was determined when p < 0.05.

**References**

1. Debalkie Animut, M., et al., *High prevalence of neurocognitive disorders observed among adult people living with HIV/AIDS in Southern Ethiopia: A cross-sectional study.* PLoS One, 2019. **14**(3): p. e0204636.

2. Zenebe, Y., et al., *Worldwide Occurrence of HIV-Associated Neurocognitive Disorders and Its Associated Factors: A Systematic Review and Meta-Analysis.* Front Psychiatry, 2022. **13**: p. 814362.

3. Grov, C., et al., *The Crisis We Are Not Talking About: One-in-Three Annual HIV Seroconversions Among Sexual and Gender Minorities Were Persistent Methamphetamine Users.* J Acquir Immune Defic Syndr, 2020. **85**(3): p. 272-279.

4. Salamanca, S.A., et al., *Impact of methamphetamine on infection and immunity.* Front Neurosci, 2014. **8**: p. 445.

5. MacDuffie, K.E., et al., *Effects of HIV Infection, methamphetamine dependence and age on cortical thickness, area and volume.* Neuroimage Clin, 2018. **20**: p. 1044-1052.

6. Soontornniyomkij, V., et al., *Effects of HIV and Methamphetamine on Brain and Behavior: Evidence from Human Studies and Animal Models.* J Neuroimmune Pharmacol, 2016. **11**(3): p. 495-510.

7. Cisneros, I.E. and A. Ghorpade, *HIV-1, methamphetamine and astrocyte glutamate regulation: combined excitotoxic implications for neuro-AIDS.* Curr HIV Res, 2012. **10**(5): p. 392-406.

8. Li, K., et al., *Reactive Astrocytes in Neurodegenerative Diseases.* Aging Dis, 2019. **10**(3): p. 664-675.

9. Wang, P. and Y. Ye, *Astrocytes in Neurodegenerative Diseases: A Perspective from Tauopathy and alpha-Synucleinopathy.* Life (Basel), 2021. **11**(9).

10. Natarajaseenivasan, K., et al., *Astrocytic metabolic switch is a novel etiology for Cocaine and HIV-1 Tat-mediated neurotoxicity.* Cell Death Dis, 2018. **9**(4): p. 415.

11. Borgmann, K. and A. Ghorpade, *Methamphetamine Augments Concurrent Astrocyte Mitochondrial Stress, Oxidative Burden, and Antioxidant Capacity: Tipping the Balance in HIV-Associated Neurodegeneration.* Neurotox Res, 2017.

12. Nooka, S. and A. Ghorpade, *Organellar stress intersects the astrocyte endoplasmic reticulum, mitochondria and nucleolus in HIV associated neurodegeneration.* Cell Death Dis, 2018. **9**(3): p. 317.

13. Borgmann, K. and A. Ghorpade, *HIV-1, methamphetamine and astrocytes at neuroinflammatory Crossroads.* Front Microbiol, 2015. **6**: p. 1143.

14. Edara, V.V., A. Ghorpade, and K. Borgmann, *Insights into the Gene Expression Profiles of Active and Restricted Red/Green-HIV(+) Human Astrocytes: Implications for Shock or Lock Therapies in the Brain.* J Virol, 2020. **94**(6).

15. Cisneros, I.E., A. Ghorpade, and K. Borgmann, *Methamphetamine Activates Trace Amine Associated Receptor 1 to Regulate Astrocyte Excitatory Amino Acid Transporter-2 via Differential CREB Phosphorylation During HIV-Associated Neurocognitive Disorders.* Front Neurol, 2020. **11**: p. 593146.

16. Proulx, J., I.W. Park, and K. Borgmann, *Cal'MAM'ity at the Endoplasmic Reticulum-Mitochondrial Interface: A Potential Therapeutic Target for Neurodegeneration and Human Immunodeficiency Virus-Associated Neurocognitive Disorders.* Front Neurosci, 2021. **15**: p. 715945.

17. Proulx, J., et al., *A Non-Canonical Role for IRE1alpha Links ER and Mitochondria as Key Regulators of Astrocyte Dysfunction: Implications in Methamphetamine use and HIV-Associated Neurocognitive Disorders.* Front Neurosci, 2022. **16**: p. 906651.

18. Shah, A., et al., *Involvement of metabotropic glutamate receptor 5, AKT/PI3K signaling and NF-kappaB pathway in methamphetamine-mediated increase in IL-6 and IL-8 expression in astrocytes.* J Neuroinflammation, 2012. **9**: p. 52.

19. Fatima, M., et al., *Novel insights into role of miR-320a-VDAC1 axis in astrocyte-mediated neuronal damage in neuroAIDS.* Glia, 2017. **65**(2): p. 250-263.

20. Priyanka, et al., *Novel role of mortalin in attenuating HIV-1 Tat-mediated astrogliosis.* J Neuroinflammation, 2020. **17**(1): p. 276.

21. Filadi, R., P. Theurey, and P. Pizzo, *The endoplasmic reticulum-mitochondria coupling in health and disease: Molecules, functions and significance.* Cell Calcium, 2017. **62**: p. 1-15.

22. Bravo, R., et al., *Endoplasmic reticulum: ER stress regulates mitochondrial bioenergetics.* Int J Biochem Cell Biol, 2012. **44**(1): p. 16-20.

23. Lebeau, J., et al., *The PERK Arm of the Unfolded Protein Response Regulates Mitochondrial Morphology during Acute Endoplasmic Reticulum Stress.* Cell Rep, 2018. **22**(11): p. 2827-2836.

24. Balsa, E., et al., *ER and Nutrient Stress Promote Assembly of Respiratory Chain Supercomplexes through the PERK-eIF2alpha Axis.* Mol Cell, 2019. **74**(5): p. 877-890 e6.

25. Nooka, S. and A. Ghorpade, *HIV-1-associated inflammation and antiretroviral therapy regulate astrocyte endoplasmic reticulum stress responses.* Cell Death Discov, 2017. **3**: p. 17061.

26. Fan, Y. and J.J. He, *HIV-1 Tat Induces Unfolded Protein Response and Endoplasmic Reticulum Stress in Astrocytes and Causes Neurotoxicity through Glial Fibrillary Acidic Protein (GFAP) Activation and Aggregation.* J Biol Chem, 2016. **291**(43): p. 22819-22829.

27. Shah, A. and A. Kumar, *Methamphetamine-mediated endoplasmic reticulum (ER) stress induces type-1 programmed cell death in astrocytes via ATF6, IRE1alpha and PERK pathways.* Oncotarget, 2016.

28. Shah, A., et al., *HIV-1 gp120 induces type-1 programmed cell death through ER stress employing IRE1alpha, JNK and AP-1 pathway.* Sci Rep, 2016. **6**: p. 18929.

29. Cisneros, I.E. and A. Ghorpade, *Methamphetamine and HIV-1-induced neurotoxicity: role of trace amine associated receptor 1 cAMP signaling in astrocytes.* Neuropharmacology, 2014. **85**: p. 499-507.

30. Zhang, Y., et al., *Involvement of sigma-1 receptor in astrocyte activation induced by methamphetamine via up-regulation of its own expression.* J Neuroinflammation, 2015. **12**: p. 29.

31. Robson, M.J., et al., *SN79, a sigma receptor antagonist, attenuates methamphetamine-induced astrogliosis through a blockade of OSMR/gp130 signaling and STAT3 phosphorylation.* Exp Neurol, 2014. **254**: p. 180-9.

32. You, Y., et al., *Activated human astrocyte-derived extracellular vesicles modulate neuronal uptake, differentiation and firing.* J Extracell Vesicles, 2020. **9**(1): p. 1706801.

33. Toussi, S.S., et al., *Short communication: Methamphetamine treatment increases in vitro and in vivo HIV replication.* AIDS Res Hum Retroviruses, 2009. **25**(11): p. 1117-21.

34. Basova, L., et al., *Dopamine and its receptors play a role in the modulation of CCR5 expression in innate immune cells following exposure to Methamphetamine: Implications to HIV infection.* PLoS One, 2018. **13**(6): p. e0199861.

35. Passaro, R.C., et al., *The Complex Interaction Between Methamphetamine Abuse and HIV-1 Pathogenesis.* J Neuroimmune Pharmacol, 2015. **10**(3): p. 477-86.

36. Luo, X. and J.J. He, *Cell-cell contact viral transfer contributes to HIV infection and persistence in astrocytes.* J Neurovirol, 2015. **21**(1): p. 66-80.

37. Lutgen, V., et al., *HIV infects astrocytes in vivo and egresses from the brain to the periphery.* PLoS Pathog, 2020. **16**(6): p. e1008381.

38. Mathe, G., et al., *Combinations of three or four HIV virostatics applied in short sequences which differ from each other by drug rotation. Preliminary results of the viral loads and CD4 numbers.* Biomed Pharmacother, 1997. **51**(10): p. 417-26.

39. Spudich, S., et al., *Persistent HIV-infected cells in cerebrospinal fluid are associated with poorer neurocognitive performance.* J Clin Invest, 2019. **129**(8): p. 3339-3346.

40. Huntington, T.E. and R. Srinivasan, *Astrocytic mitochondria in adult mouse brain slices show spontaneous calcium influx events with unique properties.* Cell Calcium, 2021. **96**: p. 102383.

41. Zhang, P., et al., *Communications between Mitochondria and Endoplasmic Reticulum in the Regulation of Metabolic Homeostasis.* Cells, 2021. **10**(9).

42. van Vliet, A.R. and P. Agostinis, *Mitochondria-Associated Membranes and ER Stress.* Curr Top Microbiol Immunol, 2018. **414**: p. 73-102.

43. Malli, R. and W.F. Graier, *IRE1alpha modulates ER and mitochondria crosstalk.* Nat Cell Biol, 2019. **21**(6): p. 667-668.

44. Ghemrawi, R. and M. Khair, *Endoplasmic Reticulum Stress and Unfolded Protein Response in Neurodegenerative Diseases.* Int J Mol Sci, 2020. **21**(17).

45. Chen, T.W., et al., *Ultrasensitive fluorescent proteins for imaging neuronal activity.* Nature, 2013. **499**(7458): p. 295-300.

46. Hayashi, T. and T.P. Su, *Sigma-1 receptor chaperones at the ER-mitochondrion interface regulate Ca(2+) signaling and cell survival.* Cell, 2007. **131**(3): p. 596-610.

47. Son, S.M., et al., *Reduced IRE1alpha mediates apoptotic cell death by disrupting calcium homeostasis via the InsP3 receptor.* Cell Death Dis, 2014. **5**: p. e1188.

48. Carreras-Sureda, A., et al., *Non-canonical function of IRE1alpha determines mitochondria-associated endoplasmic reticulum composition to control calcium transfer and bioenergetics.* Nat Cell Biol, 2019. **21**(6): p. 755-767.

49. Mori, T., et al., *Sigma-1 receptor chaperone at the ER-mitochondrion interface mediates the mitochondrion-ER-nucleus signaling for cellular survival.* PLoS One, 2013. **8**(10): p. e76941.

50. van Vliet, A.R. and P. Agostinis, *When under pressure, get closer: PERKing up membrane contact sites during ER stress.* Biochem Soc Trans, 2016. **44**(2): p. 499-504.

51. Fields, J.A., et al., *HIV alters neuronal mitochondrial fission/fusion in the brain during HIV-associated neurocognitive disorders.* Neurobiol Dis, 2016. **86**: p. 154-69.

52. Huang, C.Y., et al., *HIV-1 Vpr triggers mitochondrial destruction by impairing Mfn2-mediated ER-mitochondria interaction.* PLoS One, 2012. **7**(3): p. e33657.

53. Prasad, A., et al., *Methamphetamine functions as a novel CD4(+) T-cell activator via the sigma-1 receptor to enhance HIV-1 infection.* Sci Rep, 2019. **9**(1): p. 958.

54. Bernard-Marissal, N., et al., *Altered interplay between endoplasmic reticulum and mitochondria in Charcot-Marie-Tooth type 2A neuropathy.* Proc Natl Acad Sci U S A, 2019. **116**(6): p. 2328-2337.

55. Rosen, D.A., et al., *Modulation of the sigma-1 receptor-IRE1 pathway is beneficial in preclinical models of inflammation and sepsis.* Sci Transl Med, 2019. **11**(478).

56. Jia, J., et al., *Sigma-1 Receptor-Modulated Neuroinflammation in Neurological Diseases.* Front Cell Neurosci, 2018. **12**: p. 314.

57. Ma, R., et al., *HIV Tat-Mediated Induction of Human Brain Microvascular Endothelial Cell Apoptosis Involves Endoplasmic Reticulum Stress and Mitochondrial Dysfunction.* Mol Neurobiol, 2016. **53**(1): p. 132-142.

58. Khan, N., et al., *Involvement of organelles and inter-organellar signaling in the pathogenesis of HIV-1 associated neurocognitive disorder and Alzheimer's disease.* Brain Res, 2019. **1722**: p. 146389.

59. Gbel, J., et al., *Mitochondria-Endoplasmic Reticulum Contacts in Reactive Astrocytes Promote Vascular Remodeling.* Cell Metab, 2020. **31**(4): p. 791-808 e8.

60. Serrat, R., et al., *Astroglial ER-mitochondria calcium transfer mediates endocannabinoid-dependent synaptic integration.* Cell Rep, 2021. **37**(12): p. 110133.

61. Stalder, H., M.C. Hoener, and R.D. Norcross, *Selective antagonists of mouse trace amine-associated receptor 1 (mTAAR1): discovery of EPPTB (RO5212773).* Bioorg Med Chem Lett, 2011. **21**(4): p. 1227-31.

62. Prah, J., et al., *A novel serum free primary astrocyte culture method that mimic quiescent astrocyte phenotype.* J Neurosci Methods, 2019. **320**: p. 50-63.

63. Suzuki, J., et al., *Imaging intraorganellar Ca2+ at subcellular resolution using CEPIA.* Nat Commun, 2014. **5**: p. 4153.
